# Analytical Performance of Quantitative DiaSorin Liaison SARS-COV-2 Antigen Test for the Asymptomatic Population

**DOI:** 10.3389/fpubh.2021.788581

**Published:** 2022-01-07

**Authors:** Gema Fernández-Rivas, Jaume Barallat, Victoria Gonzalez, Silvia Martinez, Antoni E. Bordoy, Laura Jimenez, Cristina Casañ, Ignacio Blanco

**Affiliations:** ^1^Microbiology Department, Clinical Laboratory North Metropolitan Area, Germans Trias i Pujol University Hospital, Badalona, Spain; ^2^Department of Genetics and Microbiology, Autonomous University of Barcelona, Badalona, Spain; ^3^Biochemistry Department, Germans Trias i Pujol University Hospital, Badalona, Spain; ^4^Center for Epidemiological Studies on Human Immunodeficiency Virus Infection and Acquired Immunodeficiency Syndrome (HIV/AIDS) and Sexually Transmitted Infections (STI) of Catalonia (CEEISCAT), Generalitat de Catalunya, Badalona, Spain; ^5^Centro de Investigación Biomédica en Red (CIBER) in Epidemiology and Public Health (CIBERESP), Madrid, Spain; ^6^Metropolitana Nord Laboratory, Germans Trias i Pujol University Hospital, Badalona, Spain

**Keywords:** antigen detection, chemiluminescence, COVID-19 infection, asymptomatic patients, monitoring population

## Abstract

**Background:** Severe Acute Respiratory Syndrome Coronavirus 2 (SARS-CoV-2) antigen (Ag) tests have been widely employed to identify patients for a rapid diagnosis and pandemic control. Rapid lateral-flow techniques are currently the most used, but automated technologies have emerged as another viable alternative to molecular methods. We aimed to evaluate the analytical performance of the DiaSorin Liaison SARS-CoV-2 Ag test in asymptomatic population and close contacts, for its use as a tool in pandemic control efforts.

**Material and Methods:** A retrospective study was conducted. A total of 861 samples were included, 291 (34%) were positive for SARS-CoV-2 with cycle threshold (Ct) <40, and 570 (66%) were negative.

**Results:** A strong correlation was observed between reverse transcriptase-PCR (RT-PCR) Ct and Ag 50% Tissue Culture Infectious Dose per milliliter (TCID_50_/ml; *r* = 0.6486; *p* < 0.0001) and all RT-PCR negative samples tested negative for the 200 TCID_50_/ml SARS-Cov-2 Ag cutoff, i.e., a specificity of 100% was reached (95% CI: 99.4–100.0%). Samples with <25 Ct and/or >10^6^ extrapolated copies/ml were reached a sensitivity of 100% (95% IC 97.0–100.0%). For intermediate viral loads (>10^5^ extrapolated copies/ml or <30 Ct), the sensitivity value still exceeded 80%. As with other Ag methods, samples between 30 and 40 Ct could not be detected with a reliable sensitivity.

**Conclusions:** The LIAISON® SARS-CoV-2 Ag assay displays an acceptable sensitivity and a very high specificity that is useful for detecting the presence of SARS-CoV-2 in nasal swabs (NPS) of asymptomatic population or to regular monitoring of risk groups in controlled settings. Additionally, the flexibility in processing different samples and in the sampling preparation process makes this test an option for its use in high throughput laboratories. Automated tests may facilitate result reporting and yield consistent data, while avoiding some of the pitfalls of rapid lateral-flow techniques, such as observer variability.

## Introduction

Since the Coronavirus Disease-2019 (COVID-19) pandemic has emerged, efforts in its control have been focused on the development of high-sensitivity diagnostic tools and rapid systems for more afford able strategies.

The European Center for Disease Control (ECDC) in its document of November 19, 2020 “Options for the use of rapid antigen (Ag) tests for COVID-19 in the EU/EEA and the UK” indicates the need to implement policies and rapid systems of detection in certain settings where there is a high risk of transmission, such as social healthcare centers or hospital settings. The use of rapid Ag tests is appropriate in high-prevalence settings when a positive result is likely to indicate true infection and in low-prevalence settings to quickly identify highly infectious cases (1).

Given the different rapid Ag-detection tests on the market, only those that meet the WHO criteria of sensitivity (S) ≥80% and specificity (E) ≥97% and that have undergone independent validation studies (2) should be used for diagnostic purposes. Likewise, this document refers to the possibility of using these tests to monitor trends in the incidence of diseases in communities and particularly among healthcare workers (HCWs) in case of outbreaks or in areas of high community transmission where the positive and negative predictive values (NPVs) of Ag detection are enough to allow effective control policies. Rapid Ag tests were mostly developed in lateral flow devices to obtain quick results, having some limitations, such as inter-observer variability between readers or false-positive results (3). Other technologies have been developed by high throughput laboratories, minimizing reader bias and claiming to provide more consistent results than lateral flow techniques.

Antigen tests identify the presence of the nucleocapsid Severe Acute Respiratory Syndrome Coronavirus 2 (SARS-CoV-2) Ag, which is usually detectable in samples from the upper respiratory tract during the acute phase of infection. These tests do not have an amplification step, so their analytical detection limits are higher than PCR. Nonetheless, SARS-CoV-2 has an exponential growth in infected patients, so even with initial low viral loads; it is possible that in just a few hours, the viral levels reach the detection thresholds of Ag tests. This pattern of viral load kinetics could explain why repeated population screenings with Ag tests may lead to effective detection policies. Taking into account these criteria, some authors state that Ag-detection tests used frequently can still have a high sensitivity to detect carriers or infected patients without the need to meet the analytical limit of detection of the PCR as the reference test (4).

Several authors have evaluated Ag-detection tests as a screening method for a symptomatic population obtaining very good results in terms of sensitivity and specificity (5). Also, when the risk of contracting COVID-19 is lower, such as for asymptomatic individuals in low prevalence settings, the high NPVs of the Ag-detection rapid diagnostic tests (Ag-RDTs) could be useful to rule out infection. In any case, it is important to know the limitations of these tests when the option is to scale up for community and healthcare policies (6).

The option to test asymptomatic individuals has been considered to ensure a safe environment in certain settings, such as hospitals or schools (6), due to their low incidence rates. This strategy could also be boosted with the weekly performance of these tests.

Recently, the DiaSorin company has launched a SARS-CoV-2 Ag sandwich-type direct chemiluminescence immunoassay (CLIA) for a quantitative determination of SARS-CoV-2 nucleocapsid protein Ag in nasal (NS) or nasopharyngeal swabs (NPS). The final reaction consists of the emission of a light signal, giving rise to relative light units (RLU) that are directly proportional to the concentration of SARS-CoV-2 viral Ag present in the samples. Automation allows greater performance and control of the entire analytical and post-analytical process of the sample. This technology could be applied in those settings with low incidence even in an asymptomatic population or to trace COVID-19 contacts for a faster result than using PCR.

We aimed to evaluate the capacity of the LIAISON® SARS-CoV-2 Ag assay in comparison with the reference technique used for the diagnosis of SARS-CoV-2 infection (Allplex™ SARS-CoV-2 Assay, Seegene, Seoul, Korea).

## Materials and Methods

### Study Design

This retrospective study was approved by the Ethics Committee of the Germans Trias i Pujol Hospital, Badalona, Spain: PI-21-096. The study enrolled 861 asymptomatic individuals from primary healthcare centers of the Metro Nord health administrative region of Catalonia during the month of January 2021. NS specimens from these 861 individuals were collected in 3 ml Universal Transport Media (UTM) or Viral Transport Media (VTM).

The aim of this study was to assess the analytical performance of the SARS-CoV-2 Ag-detection test in asymptomatic individuals who were contacts of positive patients and in healthcare workers undergoing repeated screenings.

Asymptomatic population was defined as patients with a definitive molecular diagnosis of COVID-19 positive without presenting symptoms, such as fever, cough, myalgia, or other symptoms related to COVID-19.

Close contacts were defined as a person who has spent more than 15 consecutive minutes with the positive case at a distance closer than 2 m, within 48 h of the person getting symptoms or testing positive for SARS-CoV-2.

### Laboratory Methods

Nasal specimens were processed by reverse transcription-PCR (RT-PCR; Allplex™ SARS-CoV-2 Assay, Seegene, Seoul, Korea) according to the instructions of the manufacturer. A total of 1,000 μl of these samples were pipetted into a tube containing 1 ml of Liaison SARS-CoV-2 sample inactivation buffer for virus inactivation. The tubes were kept at room temperature for 120 min before testing. This procedure was performed on a type II biological safety cabinet. The quantification of SARS-CoV-2 Ag was determined using the Liaison SARS-CoV-2 Ag assay on the Liaison XL platform according to the instructions of the manufacturer. SARS-CoV-2 concentrations are expressed as 50% tissue culture infectious doses per milliliter (TCID_50_/ml). The Liaison XL instrument directly calculates SARS-CoV-2 viral concentrations from 22 up to 10^5^ TCID_50_/ml. The specimens with ≥200 TCID_50_/ml values are considered positive, results <200 TCID_50_/ml are considered negative. In a previous iteration of the test, results between ≥100 and <200 TCID_50_/ml were classified as equivocal. To facilitate comparisons with other authors, results have been presented with both cutoffs.

For absolute quantification based on RT-PCR cycle threshold (Ct) values, a standard curve was built using 1/2 serial dilutions of a SARS-CoV-2 RNA (Amplirun® Coronavirus RNA Control, catalog ref. MBC090, Vircell Microbiologists, Granada, Spain) in a range of concentrations from 1,400,000 copies/ml to 684 copies/ml. The measurements were made in different CFX instruments, by different technicians, and on the same day to account for the inherent variability of PCRs. The resulting standard curve was used to extrapolate the viral load of each sample (in copies/ml) from their respective Ct.

### Statistical Analysis

Concordance between results obtained for Liaison SARS-CoV-2 Ag assay and RT-PCR was established using the Spearman correlation index. In addition, NPV, positive predictive value (PPV), and positive and negative likelihood ratio (LR+, LR−) were calculated. These data were obtained with an estimated incidence of 1% according to Government Data supplied in January 2021 with RT-PCR results (Ct < 30 and Ct < 40; Tables 1A,B). The RT-PCR was used as a gold standard for the assessment of sensitivity and specificity. Statistical analyses were performed using SPSS v20 and MedCalc V19.0.6.

**Table 1:** Sensitivity, specificity, negative predictive value, positive predictive value, and positive likelihood ratio for LIAISON® SARS-CoV-2 antigen assay and (A) RT-PCR (Ct <30) and (B) RT-PCR (Ct <40) for the different cutoffs (200 TCID_50_/ml; 100 TCID_50_/ml).

**Ct = 30**	**Ag cut off 200 TCID_**50**_/mL**	**Ag cut off 100 TCID_**50**_/mL**
**(A)**		
TP	132	148
FN	570	566
FP	0	4
FN	30	14
Positive likelihood ratio	Not applicable	130.19 (48.97–346.08)
Negative likelihood ratio	0.19 (0.13–0.26)	0.09 (0.05–0.14)
VPP	100	56.8 (33.10–77.76)
VPN	99.81 (99.74–99.86)	99.91 (99.86–99.95)
**Ct = 40**	**Ag cut off 200 TCID**_**50**_ **/mL**	**Ag cut off 100 TCID**_**50**_ **/mL**
**(B)**		
TP	134	158
FN	570	566
FP	0	4
FN	157	133
Positive likelihood ratio	Not applicable	77.37 (28.97–206.61)
Negative likelihood ratio	0.54 (0.49–0.60)	0.46 (0.41–0.52)
VPP	100	43.87 (22.64–67.61)
VPN	99.46 (99.40–99.51)	99.54 (99.48–99.59)

*Ct, cycle threshold; TCID, tissue culture infectious dose per milliliter; TP, true positive; TN, true negative; FP, false positive; FN, false negative*.

## Results

**Figure 1:**
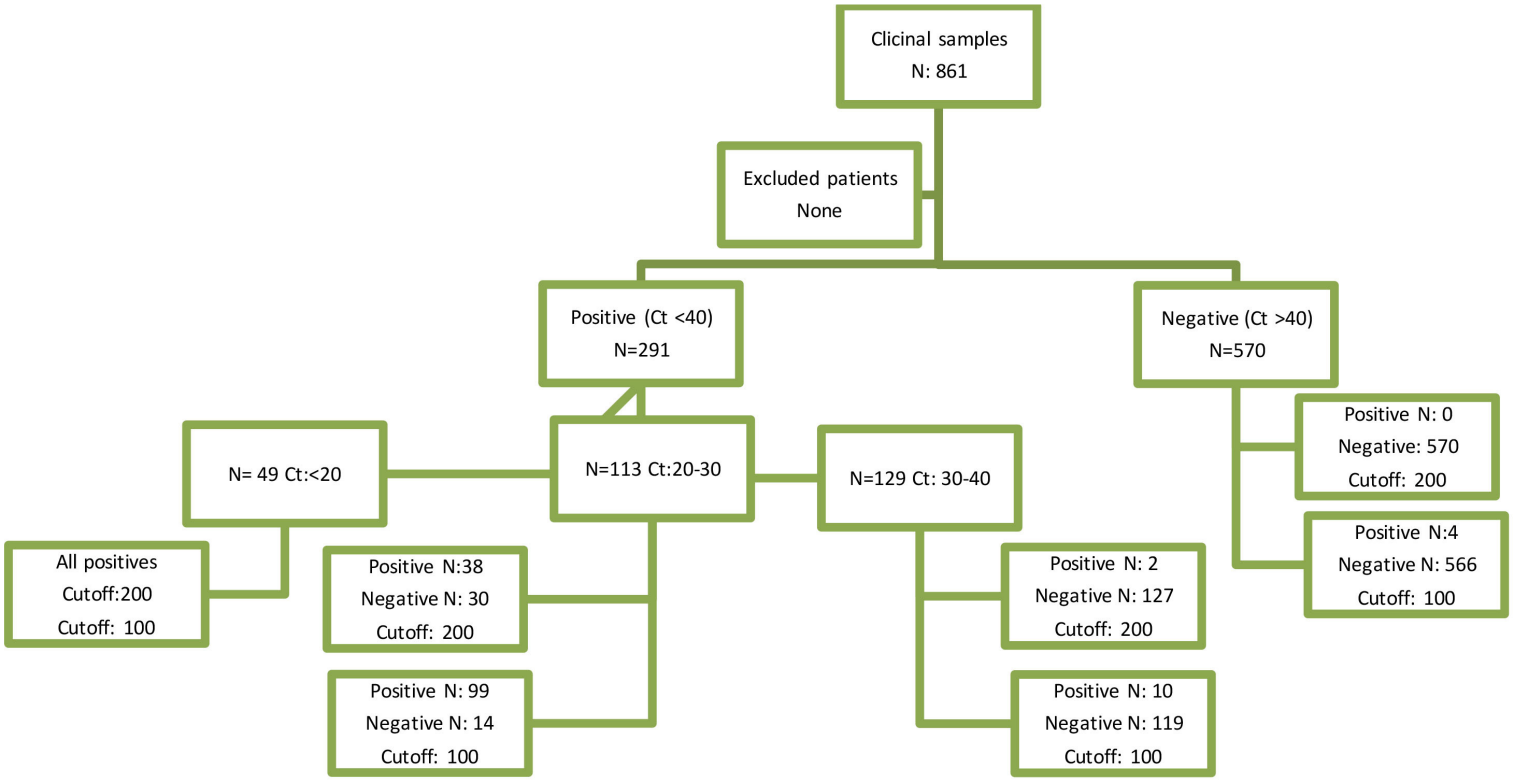
STARD diagram. STARD, standards for the reporting of diagnostic accuracy studies.

A total of 861 samples were analyzed, being 616 (72%) from female individuals, with an age distribution ranging from 1 to 94 years old (median: 44 years old). There were a total of 87 samples from patients younger than 15 years old that were included in the analysis. Two hundred and ninety-one samples (34%) were positive for SARS-CoV-2, with Ct < 40 (49 samples with a Ct < 20, 113 in a range of 20–30 Ct, and 129 in a range of 30–40 Ct; Figure 1). RT-PCR result distribution and Ag results are summarized in Table 2.

**Table 2:** Sample distribution according to Ct and Ag results by using two different cutoffs >200 TCID_50_/ml and >100 TCID_50_/ml previously classified as undetermined cutoffs as described by the manufacturer (100–199 TCID_50_/ml).

**Ct**	***n***	**Ag cut-off: 200 TCID**_**50**_ **/mL**		**Ag cut-off: 100 TCID**_**50**_ **/mL**	
		**Positive**	**Negative**	**Positive**	**Negative**
<20	49	49	0	49	0
20–24.99	62	62	0	62	0
25–27.49	28	17	11	28	0
27.5–29.99	23	4	19	9	14
30–32.49	31	2	29	8	23
32.50–34.99	40	0	40	2	38
35–40	58	0	58	0	58
Negative	570	0	570	4	566

*Ct, cycle threshold; Ag, antigen; TCID, Tissue Culture Infectious Dose per milliliter*.

**Figure 2:**
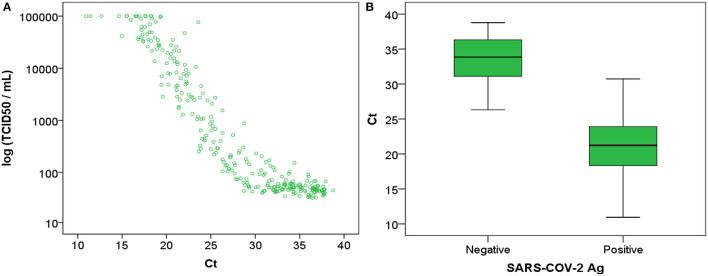
**(A)** Ag TCID_50_/ml represented against RT-PCR Ct and (**B)** RT-PCR Ct represented against antigen negative (<200 TCID_50_/ml) and positive (>200 TCID_50_/ml) results. Ct, cycle threshold; Ag, antigen; TCID, Tissue Culture Infectious Dose per milliliter.

A strong correlation was observed between RT-PCR and Ct and Ag TCID_50_/ml (*r* = 0.6486; *p* < 0.0001; Figure 2). Significant differences were observed between Ct in Ag positive and Ag negative samples with median values of 21 [interquartile range, IQR: 18–24] and 34 [IQR: 32–36], respectively.

All RT-PCR negative samples were tested negative for the 200 TCID_50_/ml cutoff SARS-CoV-2 Ag with a specificity of 100% (95% CI: 99.4–100.0%). If we consider the gray zone cutoff of 100–199 TCID_50_/ml previously proposed by the manufacturer, the specificity was 99.3% (95% CI: 98.2–99.8%). Classification differences between both cutoffs were observed only in the 25–35 Ct range. Both thresholds detected all samples with Ct < 25 and were not able to detect any sample with Ct > 35. Adjusted sensitivities for different Ct and extrapolated copies/ml are listed in Table 3. Ct-Copies extrapolation was performed using the following equation: Copies/ml = 10^14^·e^(−0.709Ct)^.

**Table 3:** Antigen determination sensitivity according to both TCID_50_/ml cutoff adjusted for (a) different Ct and (b) extrapolated copies/ml.

**Ct**	**Ag Cut off 100 TCID**_**50**_ **/mL**		**Ag Cut off 200 TCID**_**50**_ **/mL**	
	**Sensitivity**	**IC 95%**	**Sensitivity**	**IC 95%**
**(a)**				
<25	100	96.7–100.0	100	96.7–100.0
<27.5	100	96.7–100.0	92.09	86.3–96.0
<30	91.36	85.9–95.2	81.48	74.6–87.1
<32.5	80.83	74.6–86.1	69.43	62.4–75.8
<35	67.81	61.4–73.8	57.51	50.9–63.9
<40	54.3	48.4–60.1	46.05	40.2–52.0
**Copies/mL**	**Ag Cut off 100 TCID**_**50**_ **/mL**		**Ag Cut off 200 TCID50/mL**	
	**Sensitivity**	**IC 95%**	**Sensitivity**	**IC 95%**
**(b)**				
>10^6^	100	97.0–100.0	100	97.0–100.0
>10^5^	93.63	88.6–96.9	84.08	77.4–89.4
>10^4^	80.83	74.6–86.1	69.43	62.4–75.8
>10^3^	65.29	58.9–71.3	55.37	48.9–61.7
>10^2^	54.3	48.4–60.1	46.05	40.2–52.0

*TCID, Tissue Culture Infectious Dose per milliliter*.

## Discussion

One of the goals of clinical microbiology is to develop new tools for accurate diagnostic. In this sense, the COVID pandemic was a cornerstone for the introduction of molecular and new approaches for the diagnosis of infectious diseases in most clinical microbiology laboratories. The rapidity in results, such as the one offered by rapid Ag devices, is mandatory to manage the isolation of patients and to create a safe environment for healthcare workers. However, a shorter diagnosis time must not significantly reduce the ability to perform a correct diagnosis.

Rapid Ag devices, such as lateral flow tests, have many advantages but sometimes fail to detect viral Ags in specimens from which the virus was isolated and therefore pose a risk of misdiagnosing infected people. The limit of detection of Ag-RDTs could be 10,000-fold lower than those of nucleic acid amplification tests (NAATs) (7) but could be sufficient on high viral load specimens (defined as samples with real-time RT-PCR Ct values <25). Ct values do not lineally correlate with viral load and are dependent on the technique and equipment used. Moreover, it is not possible to predict viral transmissibility based only on Ct values at the individual level and results could overlap between symptomatic and asymptomatic non-spreader groups (8).

Our data suggest that for samples with a Ct value lower than 25 or >10^6^ extrapolated copies per ml, the Liaison Ag test displays a 100% of sensitivity and specificity with the currently recommended cutoff (200 TCID_50_/ml). Even with lower viral loads (>10^5^ extrapolated copies or <30 Ct), the sensitivity value is still >80% (84.08%). If the previously recommended undetermined cutoff of 100–200 TCID_50_/ml is used, a slightly better sensitivity is obtained (93.63%) for samples >10^5^ copies or <30 (91.36%) Ct, with a still high specificity of 99.3%. These data are similar to other groups who also study the performance of this technology (9, 10) but with a higher number of samples analyzed, strengthening our results.

Despite lower sensitivity compared to molecular testing, it has been postulated that Ag tests may serve as a better indicator of viral infectivity (11) and that even the increase in Ag quantitation is related to lower Ct. In this sense, the LIAISON® SARS-CoV-2 Ag assay could serve as an indicator to the evolution to the infection due to it being a quantitative technology, but more clinical and analytical studies are required to prove this hypothesis.

Likelihood ratio is used to assess how good a diagnostic test is and to help in selecting an appropriate diagnostic test. It is better than S (Sensitivity) or E (Especificity) because it is less likely to change with the prevalence of the disorder. With a 200 TCID50/ml cutoff, the LR+ is not calculable because it would have to be divided by zero, since we have not had false positives. In any case, it can be seen that the LR+ is very good in all cases and excellent at the 200 cutoff. LR– at Ct <40 is poor with both cutoffs. On the other hand, if we take 30 Ct as a cutoff point, the LR– becomes good or very good at 200 and 100 (0.54 and 0.46, respectively).

The PPV with a 200 cutoff is 100%, but on the other hand, the PPV with a cutoff of 100 is 43% or 56% depending on the Ct. Therefore, it seems clear that 200 cutoff is better for the accuracy of the test and is now the cutoff established by DiaSorin as the reference cutoff. The 100 cutoff still could be useful in populations with high suspicion of COVID-19 or high incidence rates, maintaining an indeterminate zone as a marker of follow-up.

Based on our results, values between 100 and 199 TCID_50_/ml, which could be considered equivocal, must be carefully regarded depending on clinical symptoms and the timing of possible exposure. It would be recommended that patients with equivocal results underwent several follow-up determinations to definitively rule out SARS-CoV-2 infection. As NS, oropharyngeal swabs, or even saliva samples (10) are useful for Chemiluminescence assays (CLIAs); this is a strength for an easy way for sampling and therefore, patient's adherence.

Chemiluminescence assays are commonly used in highly automated instruments and are known for their high sensitivity and practicability compared to other immunological assays (12). So, CLIA should be considered for diagnostics and control efforts in the COVID-19 pandemic. More advantages of the automated CLIA technology are the multiple types of specimens (13) and the sampling procedure. The option to deliver the sample preparation tube in the sampling settings allows saving time because while the specimens are being sent to central laboratories, the inactivation process is already taking place. This fact is not only useful for reducing time to results but also to reduce a hand-on time for the laboratory technicians, to avoid relabeling tubes, to allow a more automated process compared to other molecular systems, and to reduce the biohazard risk in laboratories. Additionally, notification of the new COVID-19 diagnostics to Public Health Authorities could also be automatic and, hence more efficient than with the lateral flow devices. Pandemic control efforts will be safer with these samples traceability. According to other authors (3), this technology could be useful in settings where controlled groups are identified and where there is a low incidence of COVID-19 infections. In these cases, a repeated sampling weekly is a cheaper and faster alternative for monitoring than molecular tests.

This study has several limitations. Firstly, our study populations are asymptomatic patients in whom the evolution of the infection is unknown, and it might be a problem because we do not work with homogeneous specimens. Additionally, some subjects develop symptoms a few days after the test, and we were not able to record this information, so some information about symptoms is not well-documented. In addition, we used frozen samples and freeze-thaw cycles may affect the integrity (14) of the N protein and hence, modify some results because small ice crystals could modify their structure, and additionally, ice could alter the concentration of proteins.

In addition, the pediatric population was included in the analysis despite it was suggested that children could be facilitators in the spread of SARS-CoV2 infection because many affected children might be asymptomatic (15) but several studies demonstrated that the frequency of asymptomatic SARS-CoV-2 carriers was similar among children and adults (16).

Lastly, the study was performed in January, so we do not know the accuracy of this test with new variants of concern; despite data demonstrate that the SARS-CoV-2 proteome is slowly accumulating mutations, fortunately (17).

In conclusion, LIAISON® SARS-CoV-2 Ag is a good alternative for diagnostic purposes for symptomatic individuals but also in close contact and asymptomatic patients. The good agreement with RT-PCR and its high specificity makes it an ideal choice as a diagnostic tool in controlled low-incidence settings. The easiness in sample management and processing is a strength for choosing LIAISON® SARS-CoV-2 Ag in the COVID-19 diagnostic algorithms.

## Data Availability Statement

The original contributions presented in the study are included in the article/supplementary material, further inquiries can be directed to the corresponding author.

## Ethics Statement

The studies involving human participants were reviewed and approved by Comitè d'Etica de la Investigació amb medicaments Hospital Universitari Germans Trias i Pujol. Written informed consent for participation was not required for this study in accordance with the national legislation and the institutional requirements.

## Author Contributions

GF-R, JB, VG, and SM: conceptualization and writing—original draft. GF-R, JB, VG, SM, and LJ: data curation. JB, VG, SM, and LJ: formal analysis. GF-R, JB, VG, SM, LJ, AB, and CC: methodology. GF-R and IB: resources. GF-R, JB, and IB: supervision. GF-R, JB, VG, SM, LJ, AB, CC, and IB: writing—review and editing. All authors contributed to the article and approved the submitted version.

## Funding

Diasorin Iberia supports this project by granting part of the funds for reagents. However, this commercial sponsor had no involvement in study design, collection, analysis, or interpretation of data, writing the manuscript, and the decision to submit the manuscript for publication.

## Conflict of Interest

The authors declare that the research was conducted in the absence of any commercial or financial relationships that could be construed as a potential conflict of interest.

## Publisher's Note

All claims expressed in this article are solely those of the authors and do not necessarily represent those of their affiliated organizations, or those of the publisher, the editors and the reviewers. Any product that may be evaluated in this article, or claim that may be made by its manufacturer, is not guaranteed or endorsed by the publisher.
